# Procedures for cryogenic X-ray ptychographic imaging of biological samples

**DOI:** 10.1107/S2052252516020029

**Published:** 2017-01-12

**Authors:** M. Yusuf, F. Zhang, B. Chen, A. Bhartiya, K. Cunnea, U. Wagner, F. Cacho-Nerin, J. Schwenke, I. K. Robinson

**Affiliations:** a London Centre for Nanotechnology, University College London, London, England; b Research Complex at Harwell, Rutherford Appleton Laboratory, Oxfordshire, England; c Division of Biosciences, Department of Life Sciences, College of Health and Life Sciences, Brunel University London, Uxbridge, England; d Department of Electrical and Electronic Engineering, Southern University of Science and Technology, Shenzhen, 518055, People’s Republic of China; e Diamond Light Source, Didcot, Oxfordshire, England; f Condensed Matter Physics and Materials Department, Brookhaven National Laboratory, Upton, NY 11973, USA

**Keywords:** human chromosomes, cell nuclei, chromosome imaging, X-ray ptychography, plunge freezing

## Abstract

Biological sample-preparation procedures have been developed for imaging human chromosomes under cryogenic conditions. A new experimental setup, developed for imaging frozen samples using beamline I13 at Diamond Light Source, is described. This manuscript describes the equipment and experimental procedures as well as the authors’ first ptychographic reconstructions using X-rays.

## Abstract 2

A new experimental setup, developed for imaging human chromosomes under cryogenic conditions using beamline I13 at Diamond Light Source, is described.

## 1. Introduction   

The high-order structure of chromosomes has been under investigation for decades. Microscopy has provided insights into the assembly of chromatin such as the 11 nm ‘beads-on-a-string’ nucleosome chain (Olins & Olins, 2003 ▸), the much debated 30 nm fibre (Finch & Klug, 1976 ▸; Robinson *et al.*, 2006 ▸; Schalch *et al.*, 2005 ▸; Song *et al.*, 2014 ▸), putative chromo­meres (Wanner & Formanek, 2000 ▸; Wanner *et al.*, 2005 ▸) and metaphase chromosomes that are in their most compact state (Nishino *et al.*, 2009 ▸; Yusuf *et al.*, 2014 ▸).

However, the processes of how the nucleosomes are organ­ized into these highly compact chromosomes in the so-called high-order structure remains to be elucidated.

For high-resolution imaging, biological samples are commonly preserved or fixed using harsh chemicals that have cross-linking effects and may not capture the true structural nature of the sample (Karnovsky, 1965 ▸). Both transmission electron microscopy (TEM) and scanning electron microscopy (SEM) are commonly used to image biological samples, but involve sample-preparation steps that can induce artifacts. These include heavy-metal staining that is used for enhancing contrast (but can precipitate), dehydration that causes shrinkage, placing samples into resins, drying with hexa­methyldisilazane (HMDS) or critical point drying (CPD) to prevent structural collapse (Weston *et al.*, 2010 ▸). On the other hand, imaging of biological samples frozen into vitrified ice, also known as cryopreservation or cryofixation, is suitable for understanding structures in as close as possible to their native state (Studer *et al.*, 2008 ▸). Common methods for freezing biological samples include high-pressure, plunge and/or slam freezing (Hurbain & Sachse, 2011 ▸). As sample thickness increases, the rate at which heat can be removed reduces. Therefore, the depth of vitrification is a principal limitation of these methods; thus, sample thickness is the main determinant of which method is most suitable (Hurbain & Sachse, 2011 ▸). In all methods, the biological samples must be frozen, with the final outcome of having vitrified ice with no ice crystals. Ice crystals cause serious imaging artifacts and their formation can severely distort the biological structures under investigation (Hurbain & Sachse, 2011 ▸; Studer *et al.*, 2008 ▸).

Even though cryo-TEM provides high resolution, samples must be thin (up to a few hundred nanometres) to allow electron penetration. Thicker samples have to be cryo­sectioned using cryo-ultramicrotomy to obtain 40–100 nm thick sections prior to imaging, which is also known as cryo-electron microscopy of vitreous sections (CEMOVIS; Al-Amoudi *et al.*, 2004 ▸; Chlanda & Sachse, 2014 ▸). This is not only time-consuming and challenging, but also involves unavoidable knife artifacts (Weston *et al.*, 2010 ▸). Automated systems such as Cryo FIB that involve sectioning using an ion beam are promising, but have only limited resolution (Schertel *et al.*, 2013 ▸). In comparison with electrons, X-rays are able to provide higher penetration, so that the sample does not have to be sectioned. Cryo-imaging has already been shown to be effective for biological samples using soft X-ray tomography (Schneider *et al.*, 2010 ▸), hard X-ray CDI (Giewekemeyer *et al.*, 2015 ▸; Huang *et al.*, 2009 ▸; Lima *et al.*, 2009 ▸, 2014 ▸; Rodriguez *et al.*, 2015 ▸) and ptychographic methods (Deng *et al.*, 2015 ▸; Lima *et al.*, 2013 ▸).

In this paper, we present our design of a cryogenic ptychography setup which uses plunge-frozen samples. The apparatus is compact, portable and flexible, as it connects to the rest of beamline I13-1 at Diamond Light Source using standard clamp-type flanges. We demonstrate the experimental workflow with our first ptychographic reconstructions of biological samples: human cell nuclei.

## 2. Materials and methods   

### 2.1. Grid preparation   

The samples used in our experiments were deposited on sample carriers for manipulation. Two type of carriers were used: silicon nitride membranes (Silson Ltd) and copper TEM grids (Taab Laboratory Equipment Ltd); both are roughly around 3 mm in external diameter. The silicon nitride membranes had an octagonal external shape and a 200 µm thick frame around a clear area of 250 × 250 µm in the centre. The TEM grids consisted of carbon-coated 7 × 7 µm quantifoils (400 mesh grids). Before sample deposition, the carriers were rendered hydrophilic either by glow-discharging them for 90 s or by dipping them in poly-l-lysine for 30 min (1:10 dilution; Boster).

### 2.2. Sample preparation   

The sample-preparation procedure followed was according to a published protocol (Shemilt *et al.*, 2015 ▸; Yusuf *et al.*, 2014 ▸). Yoruba lymphoblastoid (GM18507) cells (passage 6) were taken from liquid-nitrogen storage and cultured in fresh medium at 37°C (5% CO_2_ incubator) for 2–3 d to obtain good growth of cells. The culture medium was RPMI-1640 medium (Sigma–Aldrich, England) and was supplemented with 20% foetal bovine serum (Sigma–Aldrich) and 1% l-glutamine. The cells were treated with 2 m*M* thymidine (Sigma) and left to grow for 5 h. The cells were treated with Colcemid (Gibco BRL) at a final concentration of 0.2 µg ml^−1^ and left overnight. After hypotonic treatment (0.075 *M* KCl; VWR BDH Prolabo, Dublin, Ireland) at 37°C for 5 min, the sample was fixed in three changes of 3:1 methanol:acetic acid. Nuclei and chromosomes were validated by placing a drop onto a glass slide stained with DAPI (Sigma, catalogue No. H-1200). After placing a 22 × 50 mm cover slip, the sample was observed using a fluorescence microscope (Zeiss Z2 Axio imager with *ISIS* software). An Olympus laser scanning confocal microscope (Olympus; equipped with *LEXT* software) was also used.

Once prepared, samples were deposited by placing a drop of cell suspension on the carrier and were left to rest at room temperature for 5 min. Excess liquid was blotted away by hand using filter paper. Further blotting was performed using the built-in blotter of the plunge freezer for 1–2 s (Vitrobot, FEI). Freezing was performed in liquid ethane using the standard procedure for the instrument. Once frozen, the samples were stored in a liquid-nitrogen tank for later use.

### 2.3. Sample transfer   

The sample carriers (grids/membranes) are delicate items and may easily be damaged by direct manipulation, especially when transferred into the experimental chamber, which is under vacuum. In order to make this transfer reliable, they were mounted on a holder that can be transferred reliably and which simultaneously keeps the samples cold by increasing the thermal mass. This holder is designed to be compatible with the cryo-transfer system (VCT100, Leica) and docks in the chamber.

**Figure 1:**
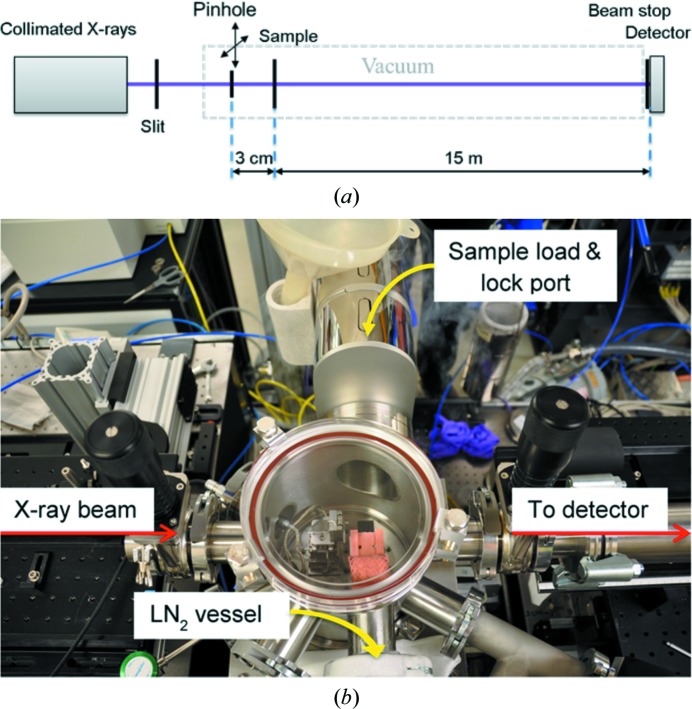
Cryo-ptychography experiment setup at I13. (*a*) Schematic of the setup and (*b*) photograph of the sample-chamber unit.

Plunge-frozen samples were transported in a dewar to the beamline. A brass transfer bucket and the sample holder were precooled under liquid nitrogen (LN_2_) in a foam bath. The loaded carriers were transferred carefully from their container onto the holder using fine cryo-tweezers and secured to the holder by a retaining ring. This operation must be performed completely submerged in liquid nitro­gen in order to preserve the sample quality. Once loaded, the holder is attached to the transfer system and introduced into the vacuum chamber following a built-in automatic pumping/venting procedure. The home-built cryo-chamber setup at beamline I13 can be seen in Fig. 1 ▸(*b*).

### 2.4. Experimental setup   

Our experiment was conducted at the coherence branch of beamline I13 at Diamond Light Source. Fig. 1 ▸(*a*) illustrates the entire setup. A pair of slits was used to generate a 0.5 × 0.5 mm wide collimated X-ray beam (9.1 keV), which was illuminated onto a 20 µm pinhole to form the probe wave on the sample placed 3 mm downstream. A Merlin detector with a pixel size of 55 µm (Quantum Detectors, UK) was located 15 m downstream of the sample and was used to acquire the diffraction patterns while the illuminiation pinhole was scanned.

To be able to cover a sample area larger than the travel range of the pinhole stage, we moved the entire chamber mounted on large translation stages. By doing so, we were able to cover an area of 5 × 5 mm, which was sufficient for the entire sample carrier. The flexible bellows attached to the two connection ports of the experimental chamber ensure constant vacuum conditions under the chamber motion.

### 2.5. Image reconstruction   

The ptychography method reconstructs a real-space image from a series of coherent diffraction patterns collected from the sample at overlapping probe positions (Maiden & Rodenburg, 2009 ▸). The degree of overlap is a critical parameter that determines the level of redundancy in the data, which in turn affects the convergence of the reconstruction algorithm (Huang *et al.*, 2014 ▸). Ptychographic data sets composed of 146 diffraction patterns were collected while the probe was scanned in a spiral pattern over a 60 × 60 µm area of the sample. The exposure time at each point was 20 s. The total data acquisition took about 50 min for one data set. A semi-transparent beam stop was used to attenuate the bright undiffracted beam so as to prevent saturation of or damage to the detector. The beamstop transmission was separately measured and was used in the re-scaling of diffraction patterns recorded with the sample. By so doing, we were able to record data with an increased dynamic range.

## 3. Results and discussion   

**Figure 2:**
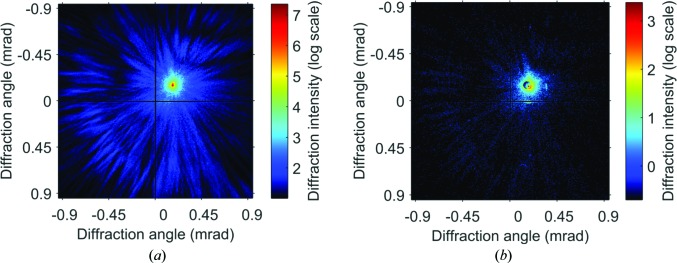
Diffraction pattern recorded for (*a*) sample and (*b*) pinhole with exposure times of 20 and 3 s. The beamstop-attenuation effect has been corrected in (*a*). The high-angle signals arose largely from the pinhole.

A typical diffraction image, after correction for the beamstop attenuation, is shown in Fig. 2 ▸(*a*) on a logarithmic scale. Although the diffraction signals spread widely to the edge of the detector, most of the high-angle diffraction arose from the illumination pinhole, as shown in Fig. 2 ▸(*b*). The pixel size in reconstruction was 86.75 nm, as calculated from the sample-to-detector distance and the detector dimensions.

**Figure 3:**
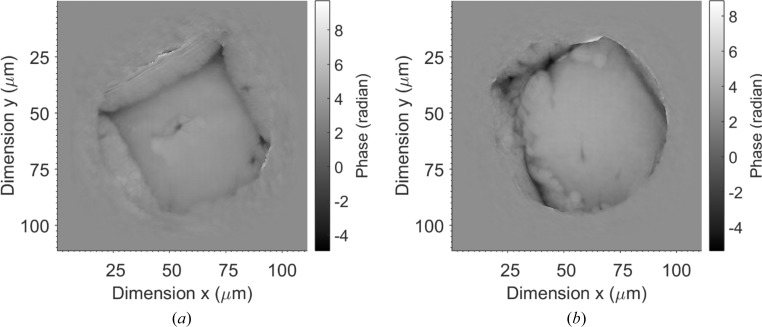
Reconstructed phase images of the nucleus sample on an Si_3_N_4_ membrane. (*a*) Fresh sample installation; (*b*) 50 min after sample installation.

The *ePIE* algorithm with positional refinement (Zhang *et al.*, 2013 ▸) was used to invert the diffraction data into images. Fig. 3 ▸ shows reconstructed phase images of the sample prepared on an Si_3_N_4_ membrane after 100 iterations of calculation for data taken sequentially from two sample regions. The measured phase corresponds to the optical path length experienced by the X-rays on travelling through the sample. The optical path length is defined as the product of the refractive index and the thickness of the sample. When the sample has a constant thickness, the phase map reflects the element distribution in the sample. In Fig. 3 ▸, only the outline of a possible nucleus can be seen; the whole sample had been covered by ice.

**Figure 4:**
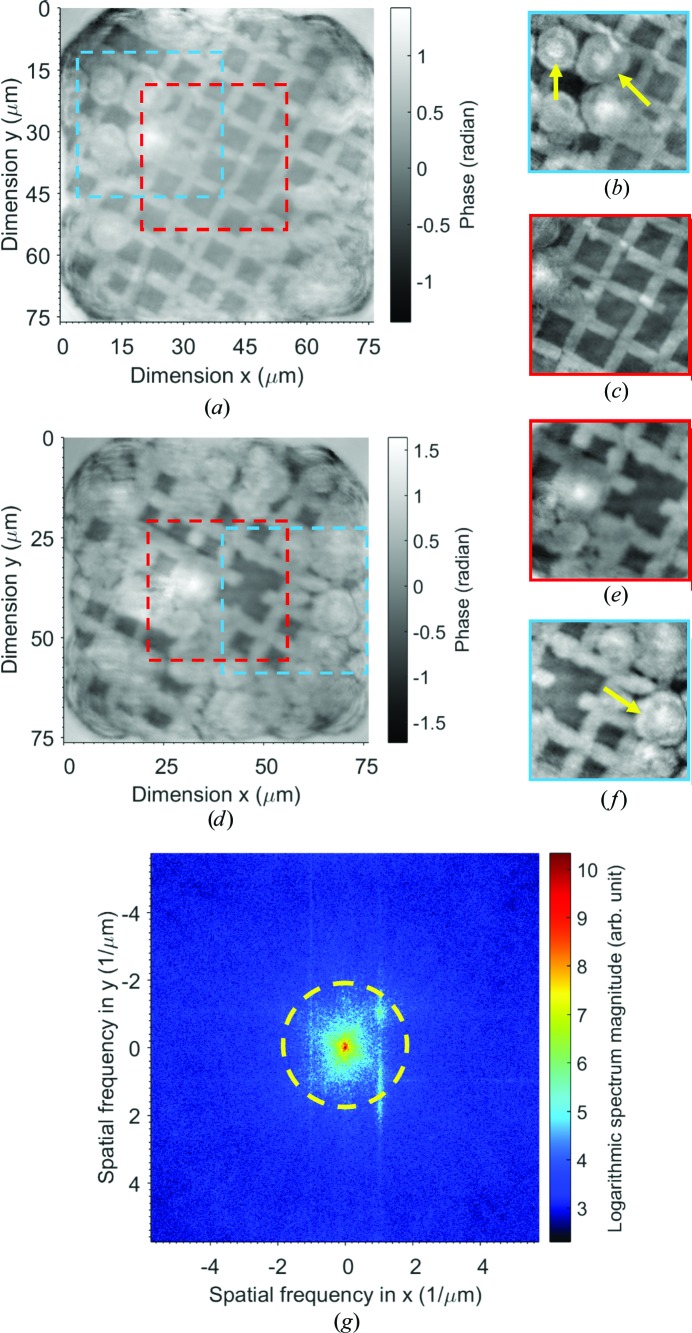
Reconstructed phase images of sample on a quantifoil TEM grid. (*a*, *b*, *c*) Fresh insertion; (*d*, *e*, *f*) 50 min after sample installation. (*b*), (*c*), (*e*) and (*f*) are reconstructions from a subarray of the recorded data; data for (*c*) and (*e*) were recorded successively in time. (*g*) The Fourier magnitude spectrum of (*c*).

A fresh sample prepared on a TEM grid was installed and the results are shown in Fig. 4 ▸. The ice covers much less of the area compared with Fig. 3 ▸, and the outline shape of the nuclei can be more clearly identified. The data for Fig. 4 ▸(*d*) were obtained about 50 min after those for Fig. 4 ▸(*a*). The growth of ice over time is evident by comparing the images. The changes in the sample during data acquisition conflict with the constant sample assumption in ptychography. With a reduced number of diffraction patterns, the image quality improves, as shown in Figs. 4 ▸(*b*) and 4 ▸(*c*) and Figs. 4 ▸(*e*) and 4 ▸(*f*). Fig. 4 ▸(*g*) shows the calculated magnitude spectrum of Fig. 4 ▸(*c*) and the dashed circle indicates a resolution of 400 nm.

Previously, human chromosomes have been analysed both optically and using X-rays (Shemilt *et al.*, 2015 ▸). To our knowledge, no studies to date have used cryogenic X-ray ptychography to image human chromosomes or nuclei. Therefore, in this demonstration study both human nuclei and chromosomes were plunge-frozen in liquid nitrogen to bring the sample into vitrified ice. It was found to be important to make the surface of the grids hydrophilic, as the chromosome sample would not settle on a hydrophobic surface. Both methods used, polylysine treatment and glow discharge, proved simple; hydrophilization with both of these methods did not show any noticeable change on making the grids hydrophilic. The most delicate part of the sample-preparation procedure was transferring the sample grid to the sample holder under liquid-nitrogen conditions. We managed to scan several samples but, with the scan time being quite long, there turned out to be a significant buildup of ice. There is a clear indication of ice buildup during each scan and with continuous scans (Figs. 3 ▸ and 4 ▸), and only nuclei-like structure can be seen in the reconstruction. No difference was seen between the two insertion types; ice was seen in both cases. The difference observed is that Fig. 3 ▸ shows a large buildup of ice upon insertion that could have been caused owing to transfer of the sample or during sample preparation, as Fig. 4 ▸ shows less ice upon insertion. This could also be owing to the samples being prepared on different inserts, with the quantifoil TEM grid having an advantage over Si_3_N_4_ membranes. The buildup of ice over the interval of measurement is also evident in the two images. It is not clear whether the hydrophilic surface aided ice growth, as we did not try hydrophobic surface samples. The formation of ice during measurement was largely from the low degree of vacuum in the sample chamber. In our setup, a large flight pipe was attached with the sample chamber and placed in the beam path to the detector. The vacuum of the flight pipe was only about 0.1 Pa, which prevented us from obtaining a higher degree of vacuum in the sample chamber. The rate of liquid-nitrogen consumption was high in the experiment, which set a maximum time for each scan of about 60 min.

One assumption in ptychography is that the wavefield exiting from the sample is the product of the sample-transmission function and the illuminating probe. The factorization of this product by the reconstruction algorithm requires that either the probe or the sample is constant at all positions whilst the diffraction patterns are recorded (Maiden & Rodenburg, 2009 ▸). In our experiments, we kept the sample stationary and scanned the illumination pinhole aperture, which differs from what has been commonly reported using a fixed probe. This arrangement solved serious technical complications arising from the cryogenic cooling of the sample while it needs to be scanned. The chosen cryogenic sample-loading system requires significant forces to be applied to the receiving stage in order to establish good thermal contact against the cold bridge. However, piezo scanning stages have only limited load capacity and driving force, and therefore their precision would be compromised by excessive mechanical loading and applied torque. In addition, the approach of scanning a pinhole was a particularly appropriate use of the long beamline of I13, which is able to provide a large coherent wave of constant amplitude and phase up to several hundred micrometres (Rau *et al.*, 2011 ▸). Cutting a probe from this wide plane wave with a scanning pinhole asserts the constant probe assumption. Additionally, variations of the probe can be taken into account in the reconstruction algorithm when necessary. Cryo-ptychography using a scanning-sample approach, which requires much more complicated hardware (Chen *et al.*, 2014 ▸; Hitchcock, 2015 ▸; Maser *et al.*, 2000 ▸), has been recently demonstrated.

Future work would be to improve the vacuum to prevent ice growth and to validate complementary samples using a cryo-fluorescence stage (Linkam) or by cryo-SEM. The sample-holder design is compatible with standard TEM grids and would allow correlative imaging with cryo-EM. The heat load in the current chamber can be reduced by a better isolation design.

## 4. Conclusions   

A cryo-ptychography experiment has been conducted on the I13 beamline of Diamond Light Source with a new particularly designed cryo-compatible vacuum chamber. A probe-scan approach was used to avoid the technical challenges in translating a cryo-sample in vacuum. Nuclei structure were revealed in the reconstruction. The low degree of vacuum caused serious ice buildup on the sample during measurements, which prevented observation of the detailed sample structure. A method of separating the sample chamber and flight pipe with a small aperture has been tested on the same beamline, which improves the vacuum by two orders of magnitude.
